# Book Review: Rheumatology in Questions

**DOI:** 10.31138/mjr.31.1.93

**Published:** 2020-03-31

**Authors:** Athanasios G. Tzioufas

**Affiliations:** Department of Pathophysiology, School of Medicine, University of Athens, Greece

Springer’s “Rheumatology in Questions” authored by Professor H.M. Moutsopoulos, Dr. E. Zampeli and Professor P.G. Vlachoyiannopoulos has recently been released, receiving excellent reviews from CNN, Forbes and Inc-Book Authority.

The book comprises 508 Questions in a total of 175 pages, organized in 14 chapters, covering almost all disciplines of systemic autoimmune rheumatic diseases, from pathogenesis to diagnostic, clinical and therapeutic issues.

The content is addressed to rheumatologists, internists, immunologists and any healthcare professional’s working in rheumatology at all training and post-qualification stages. Indeed, rheumatologists can gain insights from other fields of Internal Medicine that are invaluable, particularly for those involved in the diagnosis and management of systemic rheumatic diseases. The nature of the questions, along with the detailed, reader-friendly responses, aim towards understanding several key-points in Rheumatology, including the interpretation of immunologic tests in everyday clinical practice, the evaluation of acute phase reactants and a thoughtful presentation of the pathogenic mechanisms of these diseases in a way to comprehend: 1. Those leading to tissue injury and, eventually, damage 2. The key molecules involved in the disease process that also serve as biomarkers. 3. Molecules that are targets for treatment. On the other hand, specialists from other fields of Internal Medicine may improve their understanding of rheumatic conditions, thus enabling a better and more efficient collaboration with rheumatologists.

This manual is not meant to substitute the textbooks of rheumatology. The authors’ goal was to provide a self-evaluation tool for rheumatologists and relevant medical specialists. The view of the authors is particularly important, as the leading author, Professor and Academician H. M. Moutsopoulos, is a world leader in systemic autoimmunity. Personally, not only do I believe that they succeeded completely, but also by reading carefully the many questions and answers of this book, I realized that it provides the opportunity for the reader to be familiarized with questions generated from seasoned Rheumatology Scholars and, most importantly, to see their critical point of view from their answers.

Books like this are a significant addition to physicians’ training, as they increase the “essence of science” in the art of Medicine.

